# Performance characteristics of methods for quantifying spontaneous intracerebral haemorrhage: data from the Efficacy of Nitric Oxide in Stroke (ENOS) trial

**DOI:** 10.1136/jnnp-2014-309845

**Published:** 2015-01-09

**Authors:** Kailash Krishnan, Siti F Mukhtar, James Lingard, Aimee Houlton, Elizabeth Walker, Tanya Jones, Nikola Sprigg, Lesley A Cala, Jennifer L Becker, Robert A Dineen, Panos Koumellis, Alessandro Adami, Ana M Casado, Philip M W Bath, Joanna M Wardlaw

**Affiliations:** 1 Stroke, Division of Clinical Neuroscience, University of Nottingham, Nottingham, UK; 2 School of Pathology and Laboratory Medicine, The University of Western Australia, Nedlands, Australia; 3 Department of Medical Imaging, College of Medicine, The University of Arizona, Arizona, USA; 4 Radiological Sciences Research Group, Division of Clinical Neuroscience, University of Nottingham, Nottingham, UK; 5 Department of Neuroradiology, Nottingham University Hospitals, Queen's Medical Centre, Nottingham, UK; 6 Stroke Centre, Ospedale Sacro Cuore Via Sempreboni, Verona, Italy; 7 Division of Neuroimaging Sciences, Centre for Clinical Brain Sciences, Western General Hospital, Edinburgh, UK

**Keywords:** RANDOMISED TRIALS, STROKE

## Abstract

**Background:**

Poor prognosis after intracerebral haemorrhage (ICH) is related to haemorrhage characteristics. Along with developing therapeutic interventions, we sought to understand the performance of haemorrhage descriptors in large clinical trials.

**Methods:**

Clinical and neuroimaging data were obtained for 548 participants with ICH from the Efficacy of Nitric Oxide in Stroke (ENOS) trial. Independent observers performed visual categorisation of the largest diameter, measured volume using ABC/2, modified ABC/2, semiautomated segmentation (SAS), fully automatic measurement methods; shape, density and intraventricular haemorrhage were also assessed. Intraobserver and interobserver reliability were determined for these measures.

**Results:**

ICH volume was significantly different among standard ABC/2, modified ABC/2 and SAS: (mean) 12.8 (SD 16.3), 8.9 (9.2), 12.8 (13.1) cm^3^, respectively (p<0.0001). There was excellent agreement for haemorrhage volume (n=193): ABC/2 intraobserver intraclass correlation coefficient (ICC) 0.96–0.97, interobserver ICC 0.88; modified ABC/2 intraobserver ICC 0.95–0.97, interobserver ICC 0.91; SAS intraobserver ICC 0.95–0.99, interobserver ICC 0.93; largest diameter: (visual) interadjudicator ICC 0.82, (visual vs measured) adjudicator vs observer ICC 0.71; shape intraobserver ICC 0.88 interobserver ICC 0.75; density intraobserver ICC 0.86, interobserver ICC 0.73. Graeb score (mean 3.53) and modified Graeb (5.22) scores were highly correlated. Using modified ABC/2, ICH volume was underestimated in regular (by 2.2-2.5 cm^3^, p<0.0001) and irregular-shaped haemorrhages (by 4.8-4.9 cm^3^, p<0.0001). Fully automated measurement of haemorrhage volume was possible in only 5% of cases.

**Conclusions:**

Formal measurement of haemorrhage characteristics and visual estimates are reproducible. The standard ABC/2 method is superior to the modified ABC/2 method for quantifying ICH volume.

**Clinical trial registration:**

ISRCTN9941422.

## Introduction

Spontaneous intracerebral haemorrhage (ICH) is usually a severe form of stroke with a high mortality rate (∼50%) by the end of the first year.^1^ Poor functional outcome is related independently to increased age, systolic blood pressure, stroke severity and reduced level of consciousness. Radiologically, haemorrhage size, haemorrhage expansion, evidence of continuing bleeding (eg, ‘spot sign’ on CT angiography)^2^ ^3^ and presence of intraventricular haemorrhage (IVH) are all also associated with poor outcome.^4^ ^5^ The combination of ICH volume and initial Glasgow Coma scale was reported to be the strongest independent predictor of 30-day outcome.^1^ Hence, reliable measurement of haemorrhage size is an important component of early clinical management and for stratification in clinical trials.

Although any effective intervention will ultimately need to show an effect on functional outcome (eg, using the modified Rankin Scale (mRS))^6^ in phase III trials, earlier developments will need to study the effect of treatment on a surrogate measure. Where the intervention aims to limit haemorrhage expansion, accurate, reproducible and validated measurement of haemorrhage size (thereby allowing measurement of the change in volume) will be critical. Hence, phase II trials of blood pressure lowering and haemostatic interventions have utilised measurement of haemorrhage expansion as a key primary outcome.^7^ ^8^

There are multiple methods for measuring haemorrhage volume on a CT scan, ranging from qualitative visual estimation to computerised automatic measurement. Visual approaches (ie, visual size categorisation based on the largest diameter) are quick while computerised methods might provide a continuous volume measure but require access to good quality electronic image files. A common approach is the ABC/2 method,^9^ which has been modified such that haemorrhage slices are excluded if the area is <25% of the largest slice and counted as half if it is 25–75% of the largest slice.^10^ Alternatively, semiautomatic or fully automatic computerised methods may be used, usually based on manually outlining or thresholding areas or volumes within regions of interest (ROI). The relative advantages and disadvantages of these approaches reflect the balance between measurement time, type of data required, accuracy and availability of computer workstations, and sample size, as studied in part.^11–14^ Assessment of additional properties of haemorrhage, such as shape and density^15^ and extension into the ventricles or subarachnoid space, may also be useful.^16^ ^17^

In this study, we assessed and compared methods for measuring haemorrhage size, shape and density and IVH size. The data come from the ‘Efficacy of Nitric Oxide in Stroke’ (ENOS) trial,^18^ which assessed the management of blood pressure in acute stroke and included patients with ICH.

## Methods

### Patients

Data came from the prospective international multicentre ENOS trial, a study of blood pressure management in patients with acute ischaemic stroke or ICH, and high blood pressure.^18^ The trial had regulatory and ethics approvals in each participating country, and all patients gave written consent, or a relative provided proxy consent if the patient lacked capacity. The main inclusion criteria were acute stroke with motor deficit, systolic blood pressure of 140–220 mm Hg, and treatment could start within 48 h of onset. Patients were recruited from 173 sites in 23 countries across five continents. Following the entry of baseline demographic and clinical data into a secure internet-fronted database, patients were randomised to transdermal glyceryl trinitrate (5 mg) versus none for 7 days; those taking antihypertensive agents immediately prior to their stroke were also randomised to continue versus stop these, again for 7 days.

Participants had a baseline CT or MRI brain scan as part of clinical care, usually before randomisation. Where possible, a second (research) CT or MRI scan was performed at day 7±1 (end-of-treatment). DICOM, JPEG, PNG or GIF files are sent to the coordinating centre. Any scans sent on film were digitised using a VICOM digitiser (VIDAR Diagnostic PRO Advantage, USA). The following study assesses only those patients with a CT scan. For each patient, images were received with the thinnest slices provided by CT scan machines using standardised protocols in the recruiting centres. However, during the course of the trial, more volumetric images were made available because of advances in imaging technology.

### Scan adjudication

Complete CT scan series were made available for assessment by a group of seven adjudicators comprising accredited neuroradiologists or neurologists trained in CT brain imaging assessment in stroke (http://www.neuroimage.co.uk/sirs, with coordination by JMW).^19^ Images were viewed over the web, blind to all clinical and treatment information except for patient age, time since onset of stroke and side of brain symptoms. Responses were entered directly into the trial database via a web-based response form. Collected information included whether the patient had an ICH and its location; an estimate of its size (sorted into ordered categories based on the longest diameter in any plane): <3, 3–4.9, 5–8, >8 cm; and the presence of other qualitative findings (mass effect,^19^ atrophy,^19^ white matter disease,^20^ old infarct or haemorrhage) using validated scoring methods.

### Scan haemorrhage quantification

Scans were visualised and analysed using OsiriX for Mac (V.3, 32 bit, http://www.osirix-viewer.com^21^ on a 26 inch Apple iMac. Two trained observers (KK, SFM) measured baseline ICH volumes of 193 patients using ABC/2, modified ABC/2, semiautomatic segmentation (SAS) and automatic volume calculation (AVC, using three-dimensional (3D) rendering of a stack of two-dimensional slices). Both observers assessed scans blinded to each other's data, and repeated a proportion of the scans (47 and 34 scans for KK and SFM, respectively) at a different time blinded to their original measurements. Thus, intraobserver and interobserver variation could be estimated. For the remaining 355 patients, one observer (KK) calculated ICH volumes using all four methods.

The ABC/2 method requires measurement of the longest diameter of the haemorrhage in the axial plane (‘A’), longest axis at 90° to ‘A’ in the axial plane (‘B’), and the number of slices (‘C’) showing the haemorrhage (noting that slice thickness may vary through the scan). ‘C’ is the product of slice thickness and number of slices showing any haemorrhage. Volume is calculated as A×B×C/2.^9^ In the modified ABC/2 method, the area of haemorrhage seen on a slice has to be at least 25% of the haemorrhage area seen on the largest slice if the slice is to be considered in ‘C’; if the area is more than 75% of the largest area, the slice counts as 1, and 0.5 if the measured value of the area is between 25% and 75%.^10^

For SAS and AVC, it was possible to use thresholds so that the haemorrhage ROI was measured within certain segmentation parameters. Upper and lower attenuation values (typically 40–80 HU) of haemorrhage were established manually by sampling from the haemorrhage and normal brain. Where the threshold did not exclude non-haemorrhage areas (as with the pineal gland, calcified choroid plexus, bone) in close proximity with the haemorrhage, the haemorrhage boundary was manually edited. The area of each slice that was included within these parameters was measured, added together and multiplied by scan thickness to obtain the SAS volume. AVC used an Osirix ‘3D growing region (entire series)’ method after selecting a threshold part of the haemorrhage near to its centre. With all methods, IVH blood was included in the final ICH volume.

Additionally, four haemorrhage characteristics (area, perimeter, mean attenuation and SD of attenuation) were recorded for the haemorrhage from the slice with the largest area of haemorrhage. Haemorrhage shape and density indices were then calculated as:
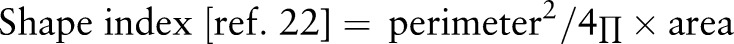


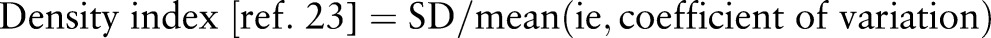


The divergence in visual appearance of haemorrhage shape and density were also assessed used an ordered categorical scale (1–5) where for an extra lesion edge or degree of density variation an additional point was given on the shape scale or density scale.^15^ Similarly, intraventricular blood volume was assessed visually using the Graeb score and modified Graeb ordered categorical scales.^16^ ^17^ The Graeb score is calculated by scoring the amount of blood in the lateral ventricles separately (score of 1=trace of blood to a maximum of 4=ventricle full of blood and expanded) and adding this to the scores for the third and fourth ventricles separately (1=blood present and 2=filled with blood and expanded) to derive a maximum score of 12. The modified Graeb score allocates scores for separate ventricular compartments (1≤25% filled with blood to 4≥75% filled with blood) to reflect selective regional accumulation of blood and an extra one point for expansion of each ventricle. The maximum score is 32.

Intraobserver and interobserver reliability was determined for visual categorisation of shape and density. In addition, intraobserver reliability was assessed for measured shape, density index, Graeb, modified Graeb and IVH volume over different reading sessions, separated by a minimum of 14 days. All adjudication and haemorrhage measurements were made blind to baseline and follow-up clinical information, other imaging and treatment assignment.

### Statistical analysis

Data are shown as number (%), median (IQR) or mean (SD). Measurement of intraobserver and interobserver variability was assessed using the intraclass correlation coefficient (ICC).^24^ A probability value of <0.05 was considered statistically significant. Analyses were performed using SPSS (V.21) and checked with Medistat running on an Apple Mac.

## Results

Altogether, 629 patients with ICH were enrolled into ENOS.^25^ ^26^ Of these, 548 patients had CT-confirmed ICH and a baseline scan available for measurement; 81 other patients either had ICH diagnosed on MRI or had no CT scan available, and these were excluded. Patient demographic and clinical details are shown in table 1. The mean age of the 548 patients in the present analysis was 67 (SD 12) years, 66% of patients were male, mean baseline blood pressure was 171 (19)/92 (13) mm Hg, and the median time from onset of ictus to performing neuroimaging was 4.5 (IQR 5.6) hours. When adjudicated visually by experts, 63% of haematomas were located in the middle cerebral artery territory (table 1); most haemorrhages caused a mass effect (86%) and many patients had leukoaraiosis (66%) and/or a previous stroke lesion (49%) present on their scans. The most frequent visually assessed haemorrhage length category was 3–5 cm (224, 41.3%), closely followed by <3 cm (220, 40.6%) with much fewer larger haemorrhages. The mean measured haemorrhage length was 3.4 cm (longest diameter; table 1).

**Table 1:** Baseline demographic, clinical and neuroradiological factors in 548 patients with primary intracerebral haemorrhage in the ENOS trial

Variable	Data
*Demographics*	
Age (years)	67.9 (12.1)
Sex, male (%)	360 (65.7)
Country, UK (%)	
Asia	117 (21.4)
Europe	67 (21.4)
Other (Africa, Australasia and North America)	42 (7.7)
UK	322 (58.8)
*Clinical findings*	
Premorbid modified Rankin Scale=0 (%)	418 (76.3)
Previous stroke (%)	69 (12.6)
Prior antihypertensive medication use (%)	227 (41.4)
Prior history of high BP (%)	341 (62.2)
Diabetes mellitus (%)	67 (12.2)
Ischaemic heart disease (%)	56 (10.2)
Atrial fibrillation (%)	30 (6.5)
Total anterior circulation syndrome (%)	195 (35.6)
SSS (/58)	30.1 (12.3)
National Institutes of Health Stroke Scale (/42)	12.8 (5.3)
Systolic BP (mm Hg)	171.6 (19.3)
Diastolic BP (mm Hg)	92.2 (13.3)
Heart rate (bpm)	77.7 (14.5)
Time, stroke to neuroimaging (h)	4.5 [11.6]
*Adjudicated CT scan findings*	
Location of haemorrhage (%)	
MCA	346 (63.1)
ACA	22 (4.0)
PCA	5 (0.9)
MCA+ACA	2 (0.4)
Borderzone	14 (2.6)
Lacunar (ie, small subcortical) stroke	142 (25.9)
Brainstem and/or cerebellum	16 (2.9)
Leukoariosis	362 (66.1)
Lesion mass effect (minimal to extreme swelling)	470 (85.9)
Previous stroke lesion	270 (49.3)
IVH	141 (25.7)
Intracerebral haematoma size category (%)	
<3 cm	220 (40.6)
3 to <5 cm	224 (41.3)
5 to 8 cm	87 (16.1)
>8 cm	11 (2.0)
*Measured CT scan findings*	
Volume, ABC/2 (cm^3^)	12.77 (16.32)
Longest diameter (cm)	3.38 (1.4)
With IVH, n=141	
Graeb score (/12)^16^	3.52 (2.4)
Modified Graeb score (/32)^17^	5.19 (4.7)
Without IVH, n=407	
Shape (/5)^15^	3.00 (1.4)
Shape index^22^	1.22 (1.1)
Density (/5)^15^	2.54 (1.3)
Density index^23^	0.19 (0.1)

Data are number (%), median (semiquartile range), or mean (SD).

NIHSS calculated from SSS scores range from 0 (comatose with quadriplegia) to 58 (normal neurological status).

ACA, anterior cerebral artery territory; BP, blood pressure; ENOS, Efficacy of Nitric Oxide in Stroke; IVH, intraventricular haemorrhage; MCA, middle cerebral artery territory; NIHSS, the National Institutes of Health Stroke Scale; PCA, posterior cerebral artery territory; SAS, semiautomatic segmentation; SSS, Scandinavian Stroke Scale.

### ICH volume

**Figure 1:**
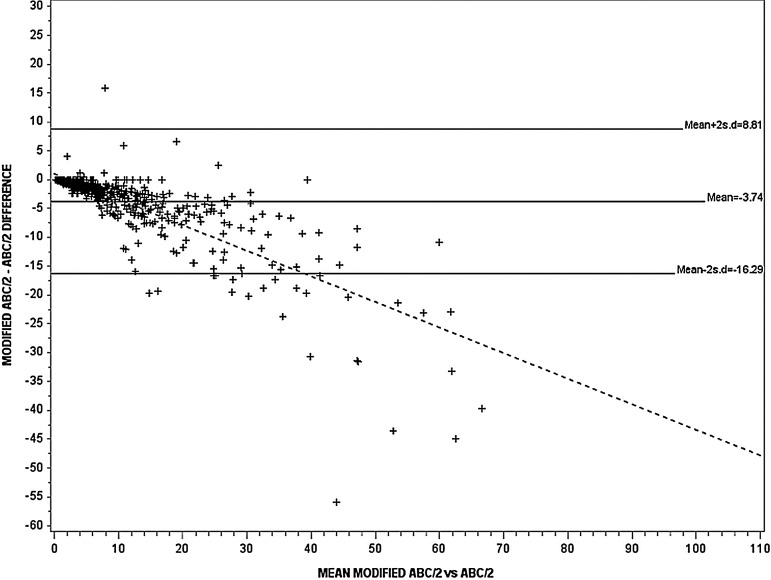
The Bland–Altman plot for assessment of variation in estimating haematoma volume between modified ABC/2 and standard ABC/2 (n=548), r^2^=0.64, p<0.0001. The continuous and dotted lines represent the regression lines. The slope of the best-fit regression line gradient was −0.44 (p<0.0001).

**Figure 2:**
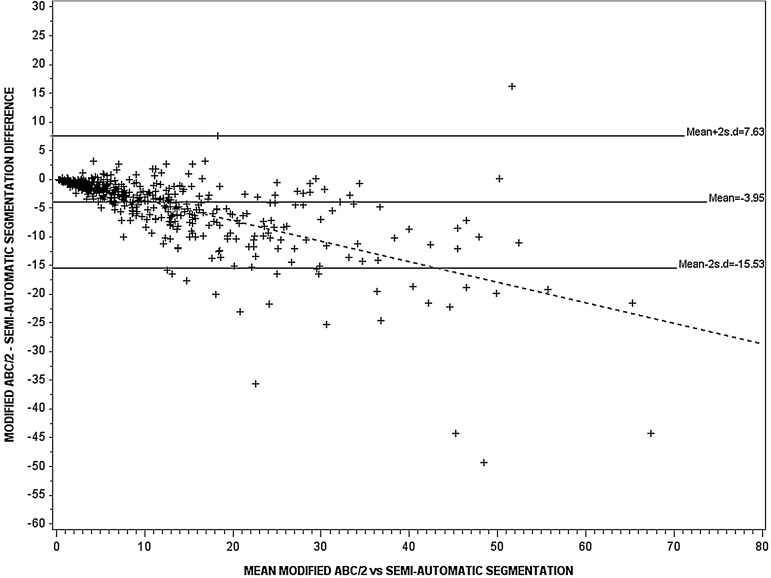
The Bland–Altman plot for assessment of variation in estimating haematoma volume between modified ABC/2 and semiautomatic segmentation (n=548), r^2^=0.45, p<0.0001. The continuous and dotted lines represent the regression lines. The slope of the best-fit regression line was −0.36 (p<0.0001).

**Figure 3:**
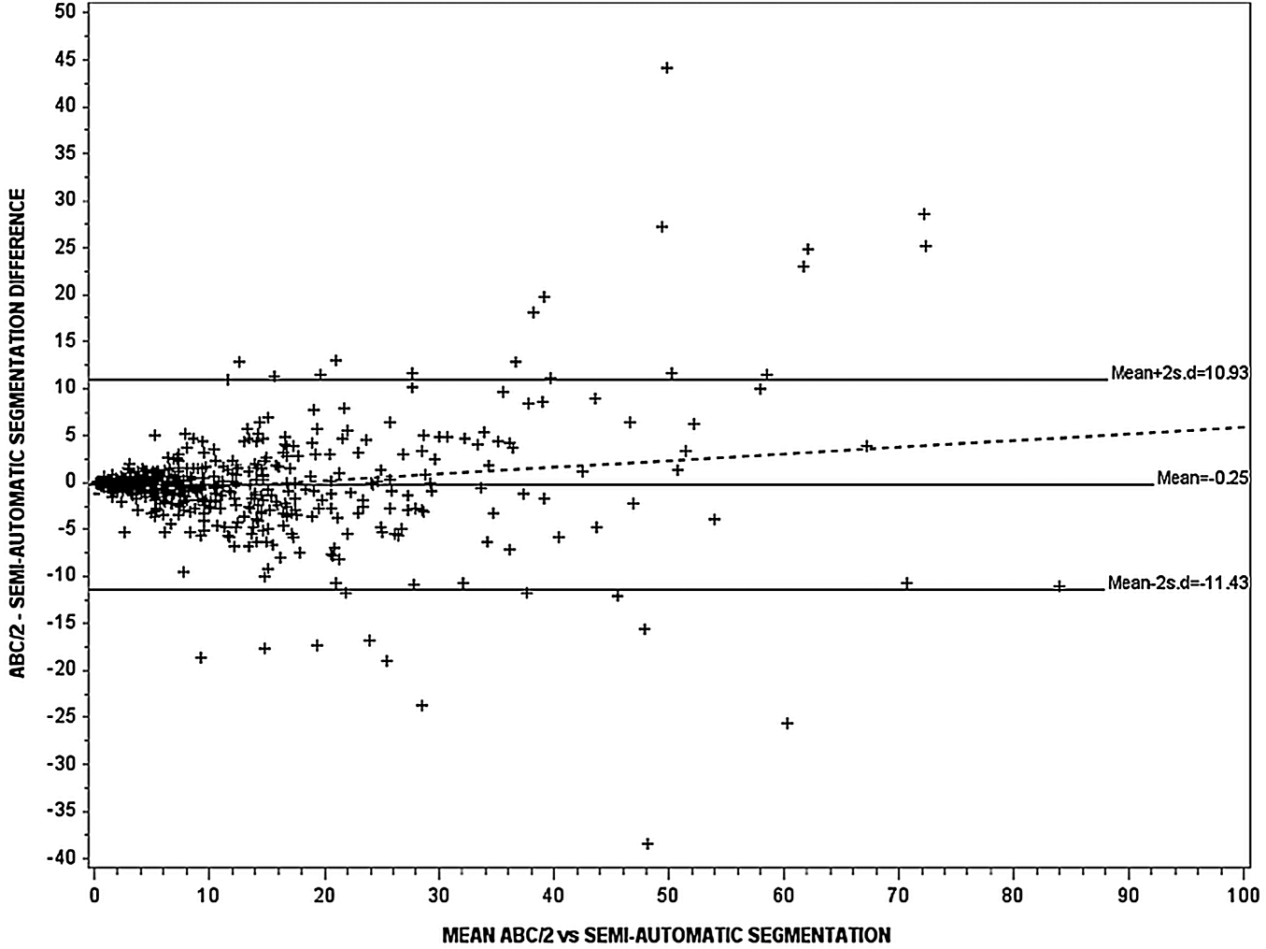
The Bland–Altman plot for assessment of variation in estimating haematoma volume (n=548) using ABC/2 and semiautomatic segmentation (n=548), r^2^=0.03, p<0.0001. The continuous and dotted lines represent the regression lines. The slope of the best-fit regression line was 0.02 (p<0.0001).

ICH volume was significantly different between ABC/2, modified ABC/2 and SAS: 12.8 (mean) (SD 16.3), 8.9 (9.2), 12.8 (13.1) cm^3^, respectively (p<0.0001) (table 2). The ICC was ‘excellent’ at 0.84–0.96 when comparing the observers’ measurements for each of ABC/2, modified ABC/2 and SAS (table 2). The observers found haemorrhage volume to be larger, by an average of 2.7–4 cm^3^ with standard ABC/2, as compared to modified ABC/2, and 1.4–4 cm^3^ smaller with SAS as compared to modified ABC/2. As the mean ICH value increased, the difference between ICH volume measured by modified ABC/2 versus the other two methods increased (figures 1 and 2), but no significant difference was observed between standard ABC/2 and SAS (table 2; figure 3). The slope of the best-fit regression line for the increasing difference between modified ABC/2 versus ABC/2 was −0.44, and −0.36 for modified ABC/2 versus SAS (both p<0.0001) (figures 1 and 2).

**Table 2:** Comparison by two observers of different methods for measuring haemorrhage volume (cm^3^) on CT scans: ABC/2 versus modified ABC/2 versus SAS

Haematoma volume		Difference	p Value	ICC
Observer 1 n=548				
ABC/2 12.77 (16.32)	Modified ABC/2 8.90 (9.21)	3.96	<0.0001	0.84
ABC/2 12.77 (16.32)	SAS 12.76 (13.06)	−0.01	1.00	
SAS 12.76 (13.06)	Modified ABC/2 8.90 (9.21)	3.97	<0.0001	
Observer 2 n=193				
ABC/2 12.05 (12.40)	Modified ABC/2 9.70 (10.05)	2.67	<0.0001	0.96
ABC/2 12.05 (12.40)	SAS 11.08 (11.38)	0.05	0.89	
SAS 11.08 (11.38)	Modified ABC/2 9.70 (10.05)	1.38	<0.0001	

Data are mean (SD), difference (Δ) in haemorrhage volume between ABC/2 and SAS and ICC.

ICC, intraclass correlation coefficient; SAS, semiautomated segmentation.

There was good intraobserver agreement for measurements assessed on 34–47 scans (5–10% of the total of 548). For ABC/2, modified ABC/2 and SAS, the intraobserver ICC was ‘excellent’, ranging between 0.97–0.98, and 0.95–0.99, respectively (table 3).

**Table 3:** Intraobserver comparison for two observers of haemorrhage size (n=47, 34 scans, respectively) on baseline CT scans from patients with ICH

			Difference (p)	ICC			Difference (p)	ICC
Observer	1				2			
Measurement	1	2			1	2		
Modified ABC/2 (cm^3^)	8.67 (8.34)	9.10 (8.34)	0.43 (0.80)	0.97	16.88 (14.60)	16.90 (14.63)	0.016 (0.97)	0.95
ABC/2 (cm^3^)	10.42 (10.28)	10.98 (10.79)	−0.55	0.97	20.97 (19.32)	20.15 (17.98)	0.82 (0.39)	0.96
SAS (cm^3^)	11.11 (10.38)	11.69 (10.99)	0.58 (0.80)	0.98	19.41 (15.32)	19.42 (15.51)	−0.11 (0.87)	0.99
Diameter (A) (cm)	3.41 (1.28)	3.37 (1.31)	0.04 (0.89)	0.98	3.89 (1.48)	4.01 (1.45)	−0.12 (0.43)	0.96

Data are mean (SD), difference in means, and ICC.

ICC, intraclass correlation coefficient; ICH, intracerebral haemorrhage; SAS, semiautomatic segmentation volume.

There was excellent interobserver agreement (ICC) based on 193 scans at 0.88 for ABC/2, 0.91 for modified ABC/2 and 0.93 for SAS (table 4). Both ABC/2 and modified ABC/2 showed excellent correlation with SAS (p<0.0001, see online supplementary figures S1–S3). Only 23 of 548 scans were amenable to analysis using the fully AVC method as the software was unable to handle the scan image with varying slice thickness; this approach was therefore ignored in further analyses.

**Table 4:** Interobserver comparison for two (observer 1 and 2; n=193 scans) of haemorrhage volume (cm^3^) on CT scans from patients with intracerebral haemorrhage (ICH)

	Observer			
	1	2	Difference (Δ)	ICC
ABC/2	10.58 (10.20)	12.05 (12.40)	−1.40	0.88
Modified ABC/2	8.42 (8.53)	9.70 (10.05)	−1.3	0.91
SAS	12.02 (12.05)	11.08 (11.38)	0.95	0.93

Data are mean (SD), difference (Δ) in haemorrhage volumes and ICC.

ICC, intraclass correlation coefficient; SAS, semiautomatic segmentation.

**Figure 4:**
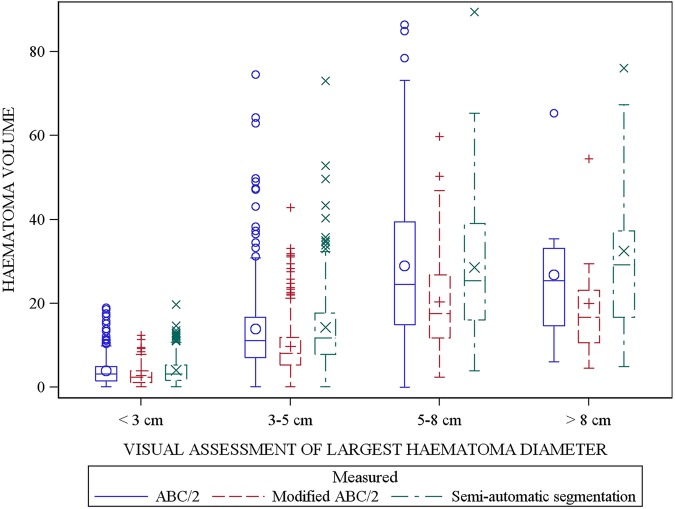
Box plots of intracerebral haemorrhage (ICH) volumes (n=548) by visually estimated size and corresponding volume measured by ABC/2, modified ABC/2 and semiautomatic segmentation (SAS). The difference between modified ABC/2 versus standard ABC/2 and modified ABC/2 versus SAS increases as the size category increases and is present in all four size categories (<3 (p<0.0001), 3–5 (p<0.001), 5–8 (p<0.0001) and >8 (p<0.0001)). There was no significant difference between standard ABC/2 and SAS in all four size categories.

Across the ICH visual size categories, ICH volumes calculated by modified ABC/2 were significantly smaller compared to SAS (figure 4). As the ICH size category increased, the difference between modified ABC/2 and SAS also increased. By comparison, there was no significant difference between ICH volumes measured by standard ABC/2 and SAS when compared by ICH visual size categorisation (figure 4).

There was good agreement between the adjudicators across ICH visual size categories (n=47, ICC 0.82, p<0.001) (see online supplementary table I) using the ordered categorical scale (<3, 3–4.9, 5–8, >8 cm)^19^ and strong agreement between the visual size category and the observer's largest measured diameter in the axial plane ‘A’ (ICC 0.71, p<0.001) (see online supplementary table II).

### ICH volume and shape

**Figure 5:**
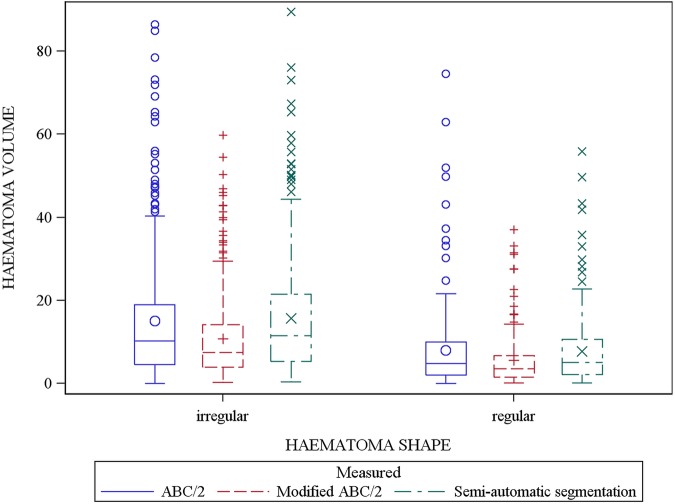
Box plots of intracerebral haemorrhage (ICH) volumes (n=548) by visually assessed shape and corresponding volume assessed by ABC/2, modified ABC/2 and semiautomatic segmentation (SAS) show: (1) larger ICHs are irregular in shape (p<0.0001); (2) the mean difference between modified ABC/2 versus standard ABC/2 and modified ABC/2 versus SAS increased as the haematoma shape became more irregular (p<0.0001). No significant difference was observed between standard ABC/2 and SAS.

The most common shape was irregular (64%), followed by regular ICH. Small ICHs (5.5–8.06 cm^3^) were more regularly shaped than larger haemorrhages (p<0.0001) (table 5). Using modified ABC/2, haemorrhage volume was significantly lower for regular-shaped haemorrhages when compared with ABC/2 (by 2.2 cm^3^) and SAS (by 2.4 cm^3^) (table 5 and figure 5); the difference was greater with larger irregular-shaped haemorrhages (between standard ABC/2 and modified ABC/2 by 4.8 cm^3^ and SAS and modified ABC/2 by 4.9 cm^3^). When compared by shape, ICH volume calculated by standard ABC/2 did not differ from that measured by SAS (figure 5).

**Table 5:** Comparison of volumes (cm^3^) using ABC/2, modified ABC/2 and semiautomated segmentation (SAS) (n=548) by haematoma shape

	Haematoma volume		Difference (Δ)	p value
Regular (1,2) n=198	ABC/2 8.06 (10.43)	Modified ABC/2 5.56 (6.31)	2.51	<0.0001
	ABC/2 8.06 (10.43)	SAS 7.77 (8.75)	0.29	0.20
	SAS 7.77 (8.75)	Modified ABC/2 5.56 (6.31)	2.21	<0.0001
Irregular (3,5) n=350	ABC/2 15.48 (18.37)	Modified ABC/2 10.66 (10.07)	4.81	<0.0001
	ABC/2 15.48 (18.37)	SAS 15.62 (14.22)	−0.14	0.82
	SAS 15.62 (14.22)	Modified ABC/2 10.66 (10.07)	4.96	<0.0001

Data are mean (SD), difference (Δ) in volume and p value.

### ICH shape and density in patients with no IVH

Intra-agreements and interagreements were both ‘good’ for visual assessments of haemorrhage shape: intraobserver ICC 0.88 (see online supplementary table III), interobserver ICC 0.75 (n=47) (see online supplementary table IV); and density: intraobserver ICC 0.86, interobserver ICC 0.73. Intra-observer ICC was 0.53 for calculated shape index and 0.86 for density index (see online supplementary table III).

### IVH volume and severity

Intraobserver agreement for assessment of IVH volume on baseline scans from 49 patients was excellent (ICC 0.98–0.99) whether using the Graeb score, modified Graeb score or SAS (see online supplementary table III). The Graeb and modified Graeb scores were highly correlated with each other (rs=0.88, p<0.01). Both were also highly correlated with measured IVH volume using the SAS method: Graeb, (rs=0.73, p<0.01); modified Graeb (rs=0.72, p<0.01).

### Comparison of ICH volume by ABC/2, modified ABC/2 to SAS

We performed a non-systematic review to compare our study with previously published work comparing ABC/2, modified ABC/2 and SAS volume measures in ICH^10–14^ ^27–30^ (see online supplementary figures S4–S7).

Some studies used the standard ABC/2 formula while others used variations of it;^10^ ^11^ Gebel *et al*^11^ used the central haemorrhage slice to measure the largest diameter ‘A’ and found higher volumes computed by ABC/2 when compared to SAS. With regard to shape, three studies studied errors in warfarin-related haemorrhage due to higher frequencies of irregular shape.^12–14^ Huttner *et al*^12^ showed that volumes of regular shaped haematomas using ABC/2 were significantly increased in regular and irregular shaped haemorrhages. However, the large slice thickness used during CT image acquisition in this study may have produced significant errors during calculation.^12^

This review comparing all three methods showed that the ABC/2 formula tended to marginally overestimate the ICH volume, but there was no absolute significant difference compared to SAS (see online supplementary figure S4); when assessed by variability in haemorrhage shape, the results follow the same sequence (see online supplementary figure S5). By comparison, ICH volumes computed using modified ABC/2 are significantly smaller when compared with SAS; the difference is greater as the haemorrhage sizes became larger and more irregular (see online supplementary figures S6 and S7).

## Discussion

To the best of our knowledge, this is the first study that compares several methods for assessing volume of spontaneous ICH and IVH volume on CT scanning and tests the effect of haematoma shape and regularity on these measures. The major findings are as follows: (1) Agreement within and between observers was excellent for measures of haemorrhage volume (ABC/2, modified ABC/2, SAS) and other haemorrhage parameters (visual categorisation by maximum length, description of shape, density and IVH); (2) haemorrhage volumes measured by modified ABC/2 were significantly lower than those measured by ABC/2 and SAS planimetry; the discrepancy increased as the haematoma size became larger; (3) larger haemorrhages were significantly more irregular in shape; (4) agreement was good between visual size categorisation and measured computerised measurement of maximum haemorrhage diameter; (5) attempts to perform a fully automated volume measurement method failed in more than 95% of patients, as the software was unable to work with scans in which slice thicknesses varied.

The sources of error that effect variation between ABC/2 and SAS merit consideration. First, the largest diameter of haemorrhage was measured in the axial plane but this may not be the largest ICH diameter, which may also have contributed to differences between ‘A’ and the visual size categorisation. Sucu *et al*^31^ suggested using the maximum length and width not necessarily on the same slice and found better correlation between ABC/2 and SAS. However, this adaptation applies only to chronic subdural haematomas which differ from spontaneous haemorrhages by extending to the cranial vault and being crescentic.^31^ Second, when using ABC/2, the scan plane is measured through AB/2, the formula for the area of a triangle. However, a triangle is not necessarily the most appropriate description of a haematoma. Last, standard ABC/2 approximates the haematoma volume as an ellipsoid with all three axes extending in three perpendicular directions^9^; to compensate for the varying slice thickness, ‘C’ is derived by multiplying the number of slices of which the haematoma is seen by the slice thickness in centimetres. For the modified ABC/2, ‘C’ does not include slices if the area of the haemorrhage is less than 25% of the largest area,^10^ an approach that has no theoretical justification. Hence, this method does not estimate all three Cartesian coordinates of the ellipsoid. Since the modified ABC/2 method underestimated the ICH volume (by 2–4 cm^3^), and small differences in volume equate to the variation in outcome,^32^ the modified ABC/2 method cannot be recommended. Further, modern CT scanners provide thin slices and the ability to directly measure ‘C’, thereby eliminating the need for approximating slice areas.^33^ ^34^

Although computer-assisted methods are considered the gold standard for volume measure,^1^ ^11^ ^12^ ^35^ the SAS method was time consuming and at times technically challenging. For instance, it was difficult to set segmentation parameters to identify a haemorrhage that was adjacent to the bone; and the threshold may miss an area of haemorrhage. Additionally, the threshold method will not account for oedema associated with the ICH, which increases the mass effect; thus, the space-occupying effect of the ICH may be much larger than its measured hyperattenuated area. As segmentation is semiautomatic, it is feasible to adjust manually for errors if they are apparent, although any adjustment may be subjective. All images require visual checking and manual correction; failure to do this would result in erroneous measurements. The problem is compounded by more irregular spontaneous haemorrhages, for example, due to amyloid, in anticoagulant-associated bleeds and in traumatic haematomas, where typically lesions are irregular, of varied attenuation and often next to the bone.

In this series of patients with spontaneous ICH, a high proportion (64%) of irregular-shaped haemorrhages were found. The effectiveness of the ABC/2 method has been validated in regular, oval-shaped haemorrhages but researchers have doubted its accuracy in complex irregular haemorrhages (eg, as seen with warfarin or in large or amyloid-related haemorrhage) and those with intraventricular extension.^12^ ^35^ It has been suggested that adjusting the denominator from 2 to 3 in ABC/2 in irregularly shaped haematomas may produce more accurate measures, but this concept is yet to be supported.^12^ ^14^ Combining the present and published findings,^29^ it may be postulated that as the haemorrhage became more irregular, the surface area and volume changes more than the largest diameter. Hence, the area of the largest haemorrhage slice may be more representative of the total haemorrhage size than its diameter. As a result, the true volume of an irregular haemorrhage may be better estimated using SAS. This finding is important since irregular shape haemorrhages are more likely to expand and affect morbidity and mortality.^15^

Our study is novel in design as it compares two commonly used methods ABC/2 and the modified formula (25%/75% distinction) with computer-assisted SAS and pragmatic visual scoring. Moreover, haemorrhage shape was incorporated in the analysis so that the source of error within each individual measurement method was assessed further. Our results show good compatibility between ABC/2 and computer-assisted SAS, irrespective of haematoma shape and poor approximation using modified ABC/2.

There are several potential explanations for variation in interobserver performance. Thinner slices may produce more accurate volume measurement,^33^ ^34^ although the scans were identical for each observer. Alternatively, each additional slice to measure increases the potential for errors. Furthermore, observers may have chosen slightly different window settings. Additionally, although the observers were all trained in using OsiriX, accuracy in measurement does depend on operator experience.^33^ ^36^

When comparing haemorrhage size on the basis of its longest measured diameter (‘A’ in ABC/2) with an adjudicated visual categorisation, there was ‘strong’ agreement, within the adjudicators and between the observers and adjudicators, with ICC 0.72. Hence, it is unsurprising that the visual category versus the largest measured axial diameter has a slightly lower agreement than some of the other measured values. Note that the visual categorisation assigns an ordinal value based on the largest size in any plane, not just the axial plane, and thus is not the same as a linear measure of largest axial diameter.

Our study also shows that qualitative descriptors of haemorrhage characteristics of haemorrhage shape, density, presence of IVH using the Graeb and modified Graeb scores can be reliably measured and are reproducible.

Of note, when measuring density heterogeneity, we used only the coefficient of variation (cv), whereas Barras used four additional measures (although these were apparently inferior to cv).^23^ Calculation of shape index using parameters obtained from the windowed haemorrhage had a low ICC of 0.53. When this result is held in context with similar computed volumes, it could be interpreted that the observer chose different threshold settings which explain the source of variability.

This study has several strengths including the use of multiple assessors, multiple methods for assessing haemorrhage characteristics, and a large data set. To minimise measurement error, observers were trained to use the OsiriX software for measuring brain CT scan parameters and in recognition of haemorrhages. Similarly, CT adjudicators were experienced neuroradiologists. Multiple observers were used to allow measurement of intraobserver and interobserver variation. The data set involved patients from five continents and so the findings have excellent external validity.

Nevertheless, the study has three significant limitations. First, the data come from a randomised controlled trial in acute stroke. Trial exclusion criteria can limit the type of patients (and therefore variation in haemorrhage) so that the data set studied here did not include patients with normal/low blood pressure, GCS<8, or without motor signs. As a result, patients with very large haematoma were under-represented. Second, the time from stroke onset to neuroimaging was relatively long (median 4.2 h), reflecting that the ENOS protocol allowed enrolment up to 48 h after stroke. Hence, ICH on later scans may already have developed some peri-ICH oedema. Finally, just over 25% of the patients had measurable IVH, and therefore the results do not represent a population where IVH is more frequent. The failure to get a fully automated volume measure in 95% of scans may be less of a problem with other software that can handle scans of variable slice thickness.

In conclusion, the modified ABC/2 formula significantly underestimates haemorrhage volume when compared to standard ABC/2 and computer assisted semiautomated segmentation (SAS) the difference increased as haematoma volume became larger and more irregular in shape. Most relevant for clinicians, our results show that the standard ABC/2 method offers more accurate quantification of ICH volume by the bedside. Although SAS is consistent and reliable, the method is slow, reliant on manual correction, advanced software and likely to cause delay in clinical decision-making often in emergency situations. Further research is required to create a faster computerised volumetric model for accurate and reliable measurement of haematomas in the clinical setting. Visual assessment using an ordered categorical scale has the strength of providing an ‘instant’ marker of haemorrhage size in the acute situation and can be applied in the absence of measurement tools and historical cut film data, and where very large study sizes or very moved images preclude computer based ABC/2 or SAS measurements. This study importantly shows that haemorrhage characteristics can be reliably measured as accuracy is pivotal to clinical trials in which ICH volume change may be a surrogate end point.

## Supplementary Material

Web supplement

Web figures
