# Supplementary material for: Performance characteristics of methods for quantifying spontaneous intracerebral haemorrhage: data from the Efficacy of Nitric Oxide in Stroke (ENOS) trial
Source: J Neurol Neurosurg Psychiatry. 2015 Jan 9;86(11):1258–66. doi: 10.1136/jnnp-2014-309845 (PMC4680163; doi:10.1136/jnnp-2014-309845)
Supplement: Web supplement [file jnnp-2014-309845-s1.pdf]

Table I. Inter-adjudicator comparison of visually estimated longest haemorrhage diameter across four size categories (<3 cm, 3-5 cm, 5-8 cm, >8 cm) in 47 patients; intra-class correlation (ICC)=0.82, p<0.001.

	Observer 2				
Observer 1	<3	3-5	5-8	>8	Total
<3	36	3	0	0	39
3-5	1	3	1	0	5
5-8	0	0	3	0	3
>8	0	0	0	0	0
Total	37	6	4	0	47

Table II. Comparison of visually estimated maximum haemorrhage category versus computerised measurement (n=548). Data are number (%), mean (standard deviation) or median[interquartile range]; intra-class correlation (ICC) = 0.71 ($p<0.001$). Modal diameter 0.59 cm.

Size category (cm)	Axial diameter 'A', minimum (cm)	Axial diameter 'A', mean (SD) (cm)	Axial diameter 'A', maximum (cm)
<3	0.66	2.22 (0.74)	5.10
3 to <5	0.59	3.76 (0.92)	7.29
5 to 8	2.12	5.07 (1.09)	7.53
>8	3.46	5.44 (1.27)	7.52
3 to <5 [<3]	0.59	3.38 (1.39)	7.53

Table III. Intra-observer comparison of haemorrhage shape, Graeb and modified-Graeb scores, and intraventricular haemorrhage. Data are mean (standard deviation) and intra-class correlation coefficient (ICC).

	Measure 1	Measure 2	ICC
Without IVH, n=82			
Shape (/5)	3.68 (1.18)	3.74 (1.14)	0.88
Shape index	1.26 (0.37)	1.24 (0.34)	0.53
Density (/5)	2.94 (1.07)	2.87 (0.99)	0.86
Density index	0.22 (0.09)	0.21 (0.08)	0.86
With IVH, n=49			
Graeb score (/12)	4.10 (2.47)	4.04 (2.43)	0.99
Modified Graeb score (/32)	6.94 (4.94)	7.04 (4.96)	0.99
Volume (by SAS, cc^3)	5.36 (8.33)	5.69 (9.32)	0.98

SAS: semi-automatic segmentation

Table IV. Inter-observer comparison of visually estimated haemorrhage shape and density. Data are mean (standard deviation) and intra-class correlation coefficient (ICC).

	Observer 1	Observer 2	ICC
N=48			
Shape (/5)	2.70 (1.23)	2.96 (1.28)	0.75, p<0.001
Density (/5)	1.62 (0.60)	1.92 (0.70)	0.73, p<0.001
