# Supplementary material for: Performance characteristics of methods for quantifying spontaneous intracerebral haemorrhage: data from the Efficacy of Nitric Oxide in Stroke (ENOS) trial
Source: J Neurol Neurosurg Psychiatry. 2015 Jan 9;86(11):1258–66. doi: 10.1136/jnnp-2014-309845 (PMC4680163; doi:10.1136/jnnp-2014-309845)

Figure 1. Graph for assessment of variation between two observers in estimating haematoma volume using ABC/2 and semi-automatic segmentation (n=193), r_2 =0.93, p<0.0001. The continuous and the dotted lines represent the regression lines. The slope of the best-fit regression line was 1.05 (p<0.0001).

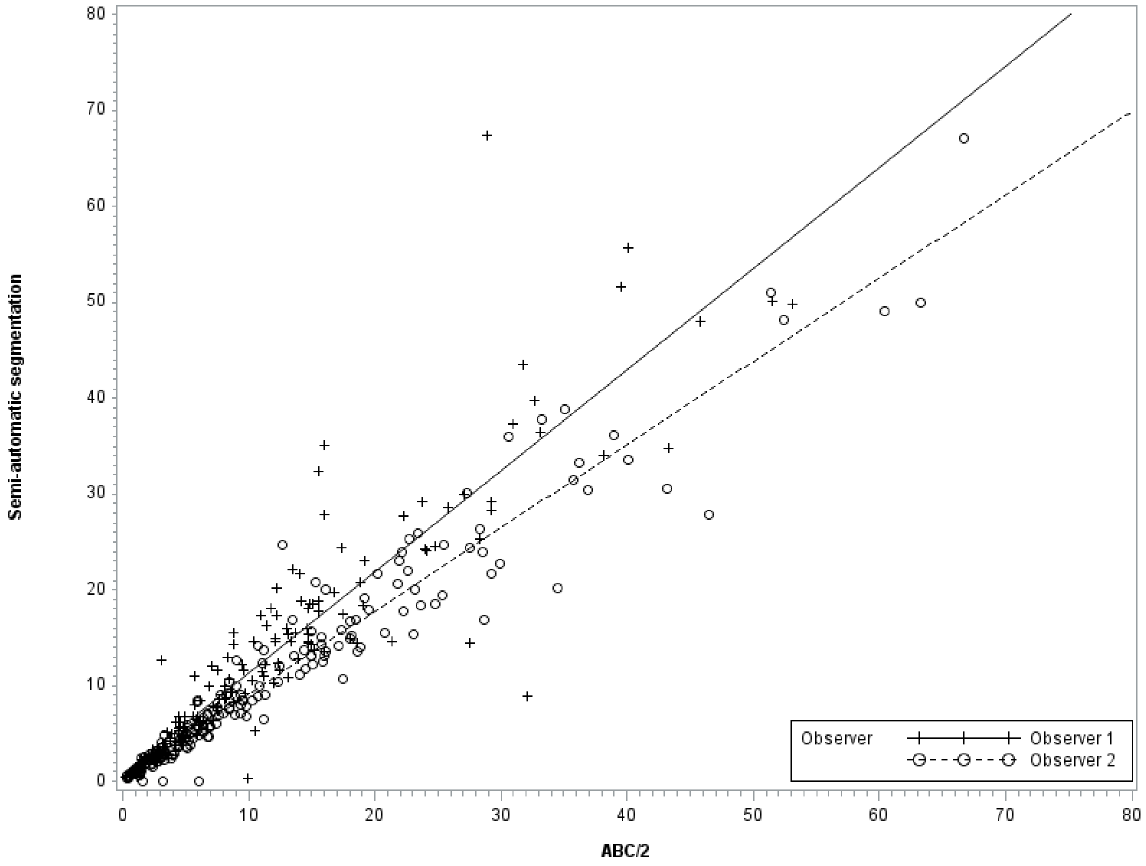

Figure 2. Graph for assessment of variation in estimating haematoma volume using ABC/2 and modified ABC/2 (n=193), $r^2 = 0.94$, $p<0.0001$. The continuous and the dotted lines represent the regression lines. The slope of the best-fit regression line was 0.78 ($p<0.0001$).

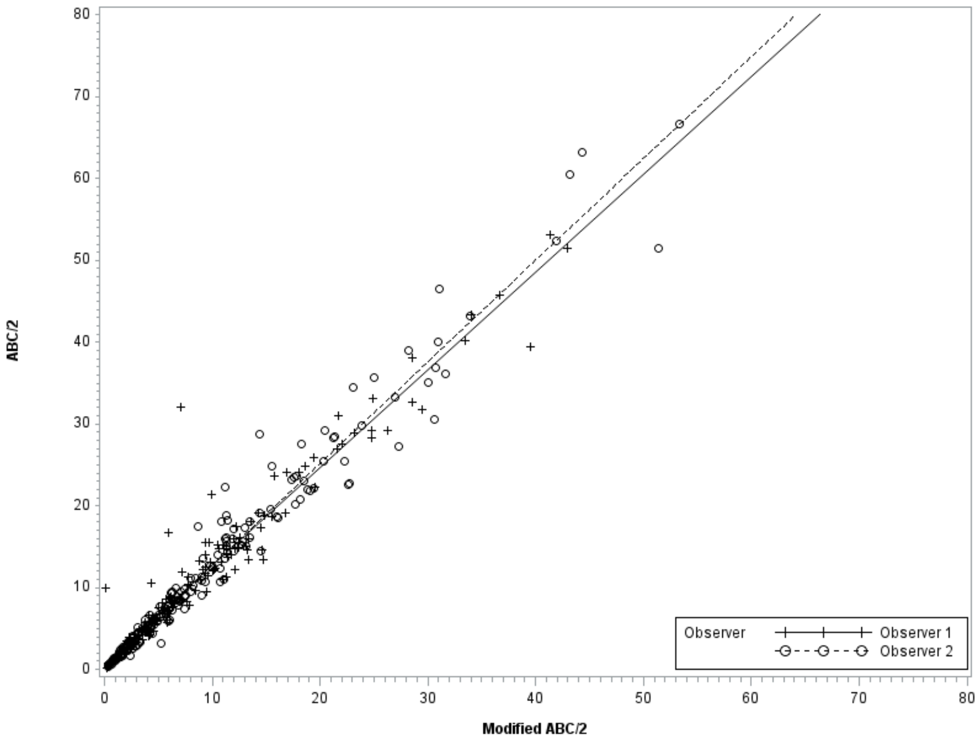

Figure 3. Graph for assessment of variation in estimating haematoma volume using modified ABC/2 and semi-automatic segmentation, r_2 =0.86, p<0.0001. The continuous and the dotted lines represent the regression lines. The slope of the bestfit regression line was 1.31 (p<0.0001).

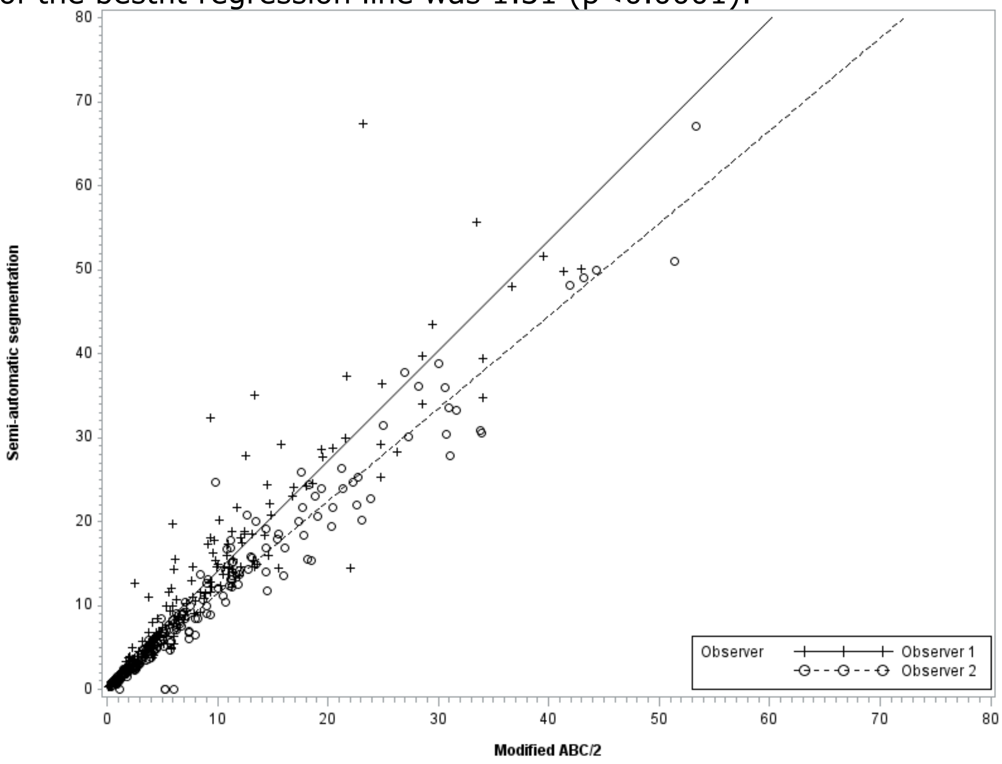

Figure 4. Forest plot of studies comparing ICH volume measurement by ABC/2 and computer- assisted SAS in spontaneous and anticoagulant related ICH. The squares indicate the point estimates and the width of the horizontal lines is the 95% confidence interval of the estimate. The diamond at the bottom represents the point estimate as well as the 95% confidence intervals of the overall effect within the categories.

	ABC/2			Planimetry				Mean Difference
**Study or Subgroup**	**Mean**	**SD**	**Total**	**Mean**	**SD**	**Total**	**Weight**	**IV, Random, 95% CI**
**1.1.1 Spontaneous ICH**								
Zhao 2009	45.98	39.97	186	43.16	37.55	186	10.4%	2.82 [-5.06, 10.70]
Yang 2013	3.67	7.04	147	4.83	7.59	147	29.8%	-1.16 [-2.83, 0.51]
Wang 2009	43.6	0	40	33.8	0	40		Not estimable
Gebel 1998	68.7	0	244	63.3	0	244		Not estimable
ENOS 2014	12.77	16.32	548	12.76	13.06	548	29.5%	0.01 [-1.74, 1.76]
**Subtotal (95% CI)**			**1165**			**1165**	**69.7%**	**-0.52 [-1.72, 0.67]**
Heterogeneity: $Tau^2 = 0.00$; $Chi^2 = 1.60$, df = 2 (P = 0.45); $I^2 = 0\%$								
Test for overall effect: Z = 0.86 (P = 0.39)								
**1.1.2 Anticoagulation ICH**								
Huttner 2006	40.83	3.9	50	36.6	3.5	45	30.3%	4.23 [2.74, 5.72]
**Subtotal (95% CI)**			**50**			**45**	**30.3%**	**4.23 [2.74, 5.72]**
Heterogeneity: Not applicable								
Test for overall effect: Z = 5.57 (P < 0.00001)								
**1.1.3 Spontaneous ICH vs Anticoagulation ICH**								
**Subtotal (95% CI)**			**0**			**0**		**Not estimable**
Heterogeneity: Not applicable								
Test for overall effect: Not applicable								
**Total (95% CI)**			**1215**			**1210**	**100.0%**	**1.23 [-1.85, 4.31]**
Heterogeneity: $Tau^2 = 7.57$; $Chi^2 = 25.42$, df = 3 (P < 0.0001); $I^2 = 88\%$								
Test for overall effect: Z = 0.78 (P = 0.43)								
Test for subgroup differences: $Chi^2 = 23.81$, df = 1 (P < 0.00001), $I^2 = 95.8\%$								

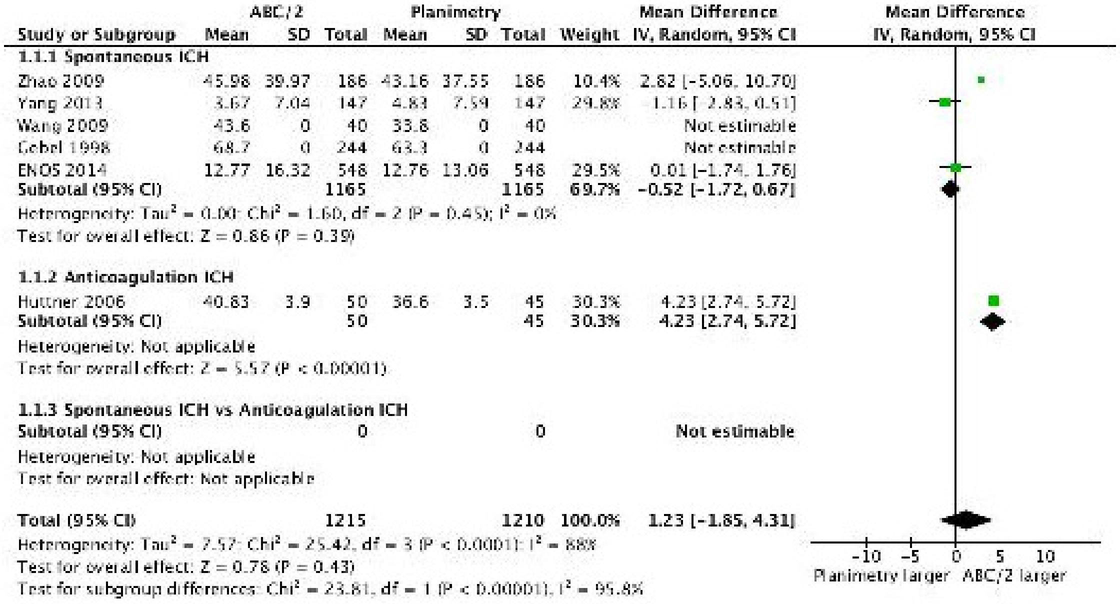

Figure 5. Forest plot of studies comparing variation between ABC/2 and computerassisted SAS measurements by haematoma shape. The squares indicate the point estimates and the width of the horizontal lines is the 95% confidence interval of the estimate. The diamond at the bottom represents the point estimate as well as the 95% confidence intervals of the overall effect within the categories.

	ABC/2 (original)			Planimetry				Mean Difference
**Study or Subgroup**	**Mean**	**SD**	**Total**	**Mean**	**SD**	**Total**	**Weight**	**IV, Random, 95% CI**
**1.2.1 Regular**								
ENOS 2014	8.06	10.43	198	7.77	8.75	198	15.6%	0.29 [-1.61, 2.19]
Huttner 2006	42.5	6.6	28	39.9	4.8	28	12.5%	2.60 [-0.42, 5.62]
Yang 2013	2.37	3.25	51	3.03	4.57	51	16.5%	-0.66 [-2.20, 0.88]
**Subtotal (95% CI)**			**277**			**277**	**44.6%**	**0.34 [-1.25, 1.92]**
Heterogeneity: $Tau^2 = 0.87$; $Chi^2 = 3.60$, df = 2 (P = 0.17); $I^2 = 44\%$								
Test for overall effect: Z = 0.42 (P = 0.68)								
**1.2.2 Irregular**								
ENOS 2014	15.48	18.37	350	15.62	14.22	350	14.1%	-0.14 [-2.57, 2.29]
Huttner 2006	38.9	8.2	28	34.3	6.6	28	10.3%	4.60 [0.70, 8.50]
Yang 2013	5.47	9.06	96	6.14	8.78	96	13.9%	-0.67 [-3.19, 1.85]
**Subtotal (95% CI)**			**474**			**474**	**38.3%**	**0.88 [-1.84, 3.59]**
Heterogeneity: $Tau^2 = 3.55$; $Chi^2 = 5.33$, df = 2 (P = 0.07); $I^2 = 63\%$								
Test for overall effect: Z = 0.63 (P = 0.53)								
**1.2.3 Seperated**								
Huttner 2006	43.3	9.7	27	32.9	7.9	27	8.5%	10.40 [5.68, 15.12]
Yang 2013	9.79	8.33	21	9.75	7.07	21	8.6%	0.04 [-4.63, 4.71]
**Subtotal (95% CI)**			**48**			**48**	**17.1%**	**5.21 [-4.94, 15.37]**
Heterogeneity: $Tau^2 = 47.92$; $Chi^2 = 9.35$, df = 1 (P = 0.002); $I^2 = 89\%$								
Test for overall effect: Z = 1.01 (P = 0.31)								
**Total (95% CI)**			**799**			**799**	**100.0%**	**1.51 [-0.36, 3.37]**
Heterogeneity: $Tau^2 = 4.86$; $Chi^2 = 26.35$, df = 7 (P = 0.0004); $I^2 = 73\%$								
Test for overall effect: Z = 1.58 (P = 0.11)								
Test for subgroup differences: $Chi^2 = 0.94$, df = 2 (P = 0.63), $I^2 = 0\%$								

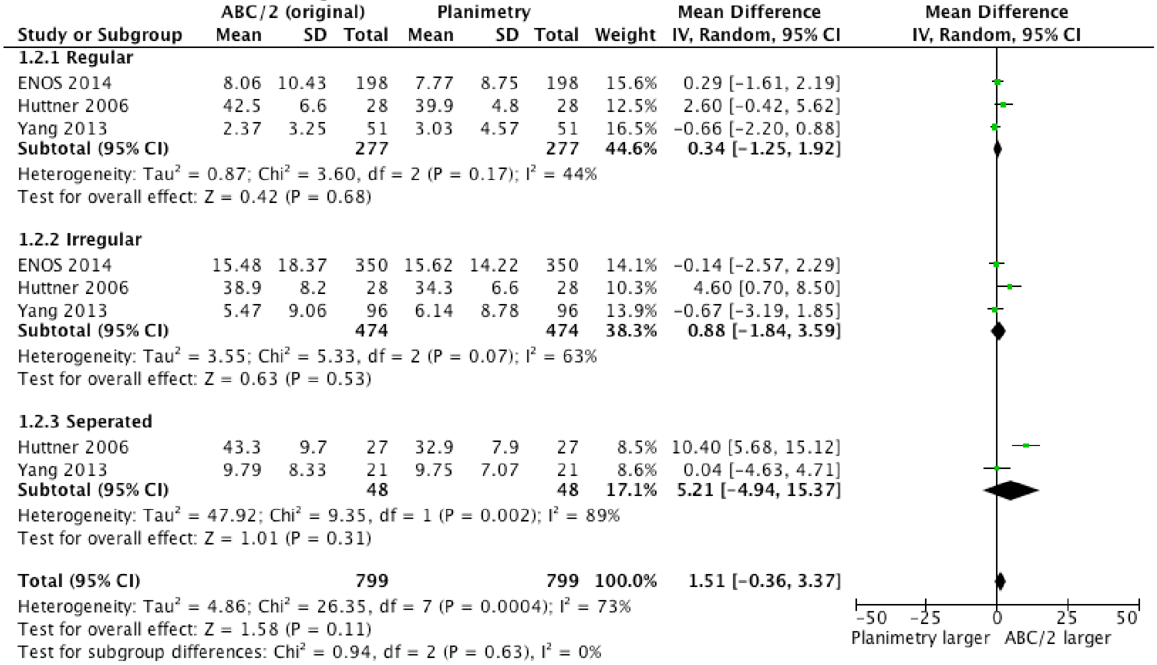

Figure 6. Forest plot of studies comparing ICH volume by modified ABC/2 and computer-assisted SAS in spontaneous and anticoagulation related ICH. The squares indicate the point estimates and the width of the horizontal lines is the 95% confidence interval of the estimate. The diamond at the bottom represents the point estimate as well as the 95% confidence intervals of the overall effect within the categories.

3.1 ABC/2 (modified) vs planimetry

	ABC/2 (modified)			Planimetry				Mean Difference
**Study or Subgroup**	**Mean**	**SD**	**Total**	**Mean**	**SD**	**Total**	**Weight**	**IV, Random, 95% CI**
**3.1.1 Spontaneous ICH**								
ENOS 2014	8.8	9.21	548	12.76	13.06	548	33.3%	-3.96 [-5.30, -2.62]
Kosior 2011	48	41.6	30	33.1	26.1	30	5.1%	14.90 [2.67, 32.47]
Kothari 1996	27.5	2.9	118	26	2.6	118	34.1%	1.50 [0.80, 2.20]
Maeda 2013	12.8	11.37	20	15.04	12.46	20	17.0%	2.24 [9.63, 5.15]
Sheth 2010	30	37	50	35	38	50	6.8%	-5.00 [-19.70, 9.70]
**Subtotal (95% CI)**			**766**			**766**	**96.3%**	**-0.80 [-5.19, 3.59]**
Heterogeneity: $Tau^2 = 14.09$; $Chi^2 = 53.74$, df = 4 (P < 0.00001); $I^2 = 93\%$								
Test for overall effect: Z = 0.36 (P = 0.72)								
**3.1.2 Anticoagulation ICH**								
Freeman 2008	72.2	0	7	75.4	0	7		Not estimable
Sheth 2010	44	51	50	53	56	50	3.7%	9.00 [29.99, 11.99]
**Subtotal (95% CI)**			**57**			**57**	**3.7%**	**-9.00 [-29.99, 11.99]**
Heterogeneity: Not applicable								
Test for overall effect: Z = 0.84 (P = 0.40)								
**Total (95% CI)**			**823**			**823**	**100.0%**	**-1.11 [-5.40, 3.19]**
Heterogeneity: $Tau^2 = 13.97$, $Chi^2 = 54.50$, df = 5 (P < 0.00001); $I^2 = 91\%$								
Test for overall effect: Z = 0.51 (P = 0.61)								
Test for subgroup differences: $Chi^2 = 0.56$, df = 1 (P = 0.45), $I^2 = 0\%$								

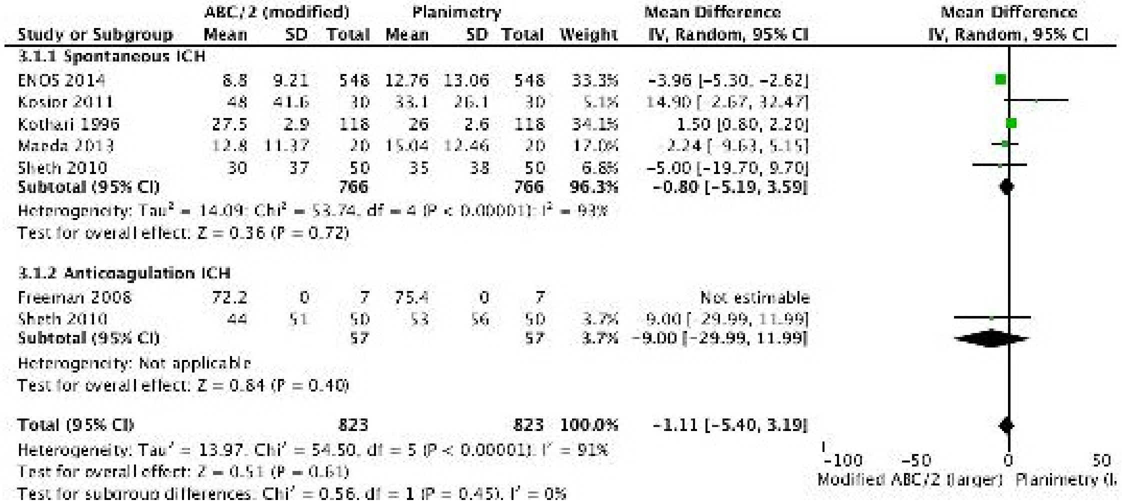

Figure 7. Forest plot of studies comparing variation between modified ABC/2 and computer-assisted SAS by haematoma shape. The squares indicate the point estimates and the width of the horizontal lines is the 95% confidence interval of the estimate. The diamond at the bottom represents the point estimate as well as the 95% confidence intervals of the overall effect within the categories.

	ABC/2 (modified)			Planimetry				Mean Difference
Study or Subgroup	Mean	SD	Total	Mean	SD	Total	Weight	IV, Random, 95% CI
**3.2.1 Regular**								
ENOS 2014	5.56	6.31	198	7.77	8.75	198	51.8%	−2.21 [−3.71, −0.71]
Freeman 2008	22	0	3	28.7	0	3		Not estimable
**Subtotal (95% CI)**			**201**			**201**	**51.8%**	**−2.21 [−3.71, −0.71]**
Heterogeneity: Not applicable								
Test for overall effect: Z = 2.88 (P = 0.004)								
**3.2.2 Irregular**								
ENOS 2014	10.66	10.07	350	15.62	14.22	350	48.2%	−4.96 [−6.79, −3.13]
Freeman 2008	110	0	4	110.5	0	4		Not estimable
**Subtotal (95% CI)**			**354**			**354**	**48.2%**	**−4.96 [−6.79, −3.13]**
Heterogeneity: Not applicable								
Test for overall effect: Z = 5.33 (P < 0.00001)								
**Total (95% CI)**			**555**			**555**	**100.0%**	**−3.53 [−6.23, −0.84]**

Heterogeneity: $Tau^2 = 3.05$; $Chi^2 = 5.20$, df = 1 (P = 0.02); $I^2 = 81\%$
Test for overall effect: Z = 2.57 (P = 0.01)
Test for subgroup differences: $Chi^2 = 5.20$, df = 1 (P = 0.02), $I^2 = 80.8\%$

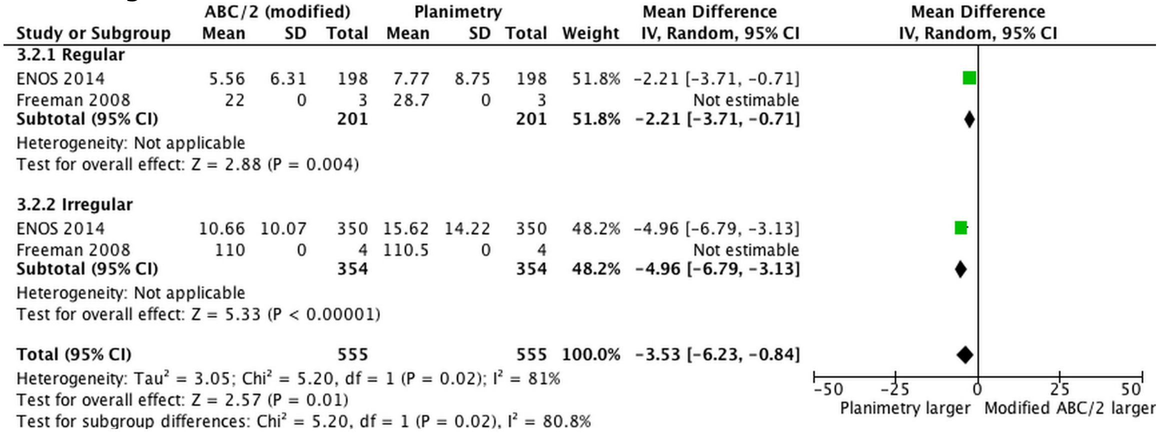

Supplement: Web figures [file jnnp-2014-309845-s2.pdf]
